# Supplementary material for: S100A8/A9 inhibition reduces splenic myelopoiesis and improves outcomes after stroke
Source: Front Immunol. 2026 Feb 17;17:1768647. doi: 10.3389/fimmu.2026.1768647 (PMC12953063; doi:10.3389/fimmu.2026.1768647)
Supplement: Supplementary file 1 [file DataSheet1.pdf]

Supplementary Material

S100A8/A9 Inhibition Reduces Splenic Myelopoiesis and Improves Outcomes After Stroke

Hyun Ah Kim[1,2]†, Annas Al-sharea[3]†, Hannah X. Chu[1,2]†, Sung-Chun Tang[4], Samoda A. Rupasinghe[1,2], Shenpeng R. Zhang[1,2], Prabhakara R Nagareddy[5], Grant R. Drummond[1,2], Thiruma V. Arumugam[1,2], Andrew J. Murphy[3,6,7], Christopher G. Sobey[1,2]*, Man K.S. Lee[3,6,7,8].

[1]Centre for Cardiovascular Biology and Disease Research, La Trobe Institute for Molecular Sciences, La Trobe University, Bundoora, VIC, Australia.

[2]Department of Microbiology, Anatomy, Physiology and Pharmacology, School of Agriculture, Biomedicine and Environment, La Trobe University, Bundoora, VIC, Australia.

[3]Haematopoiesis and Leukocyte Biology, Baker Heart and Diabetes Institute, Melbourne, VIC, Australia.

[4] Stroke Center, Department of Neurology, National Taiwan University Hospital, Taipei, Taiwan.

[5]Department of Internal Medicine, Section of Cardiovascular Diseases, University of Oklahoma Health Sciences Center (OUHSC), Oklahoma City, OK, USA.

[6]Baker Department of Cardiometabolic Health, The University of Melbourne, Melbourne, VIC, Australia.

[7]Department of Diabetes, Monash University, Melbourne, VIC, Australia.

[8]Baker Department of Cardiovascular Research, Translation and Implementation, La Trobe University, Bundoora, VIC, Australia.

†These authors contributed equally to this work and share first authorship

*Corresponding author

Number of Supplementary Figures: 4

Supplementary Figure 1

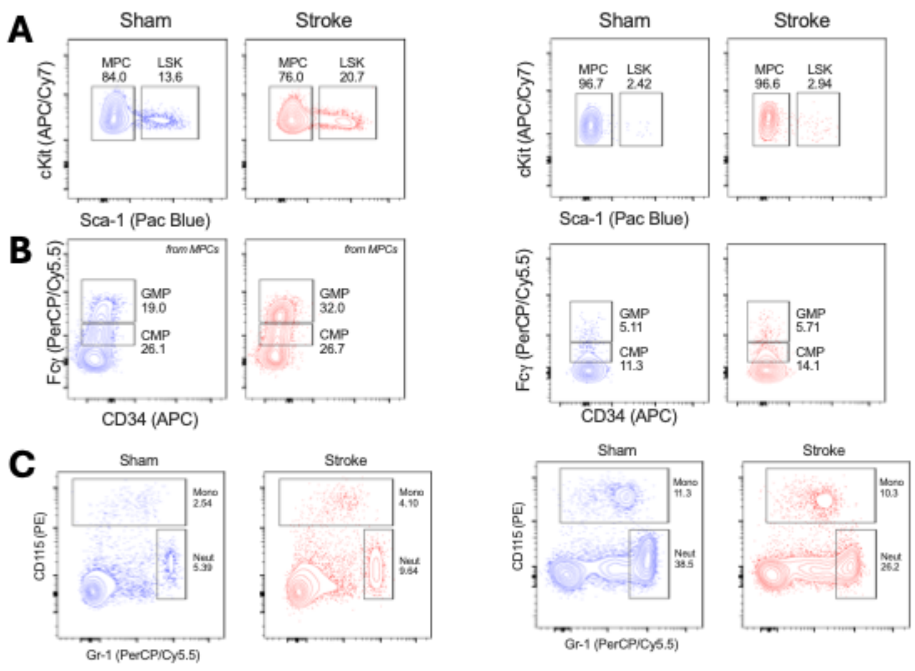

Flow cytometry gating strategy.

(A) Representative gating strategy for LSKs (lineage−/Sca-1+/c-Kit+) in bone marrow and spleen. **(B)** Gating for common myeloid progenitors (CMPs) and granulocyte–monocyte progenitors (GMPs). **(C)** Gating for mature neutrophils and monocytes.

Supplementary Figure 2

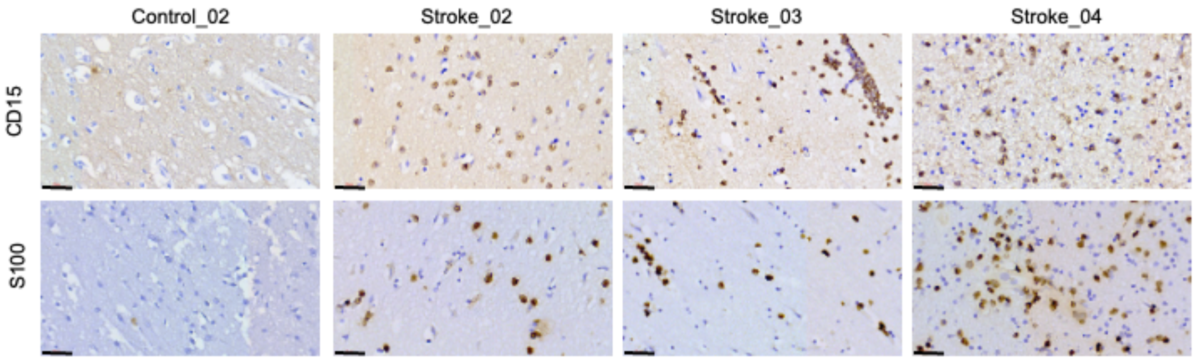

S100A8/A9 and neutrophils in human post-mortem stroke brain. Expression of S100A8/A9 and neutrophils in human post-mortem brain from a non-stroke subject and three stroke patients. These are in addition to patients presented in Figure 1 (i.e. data comprise n=2 control and n=4 stroke brains). Scale bar = 50 µm.

Supplementary Figure 3

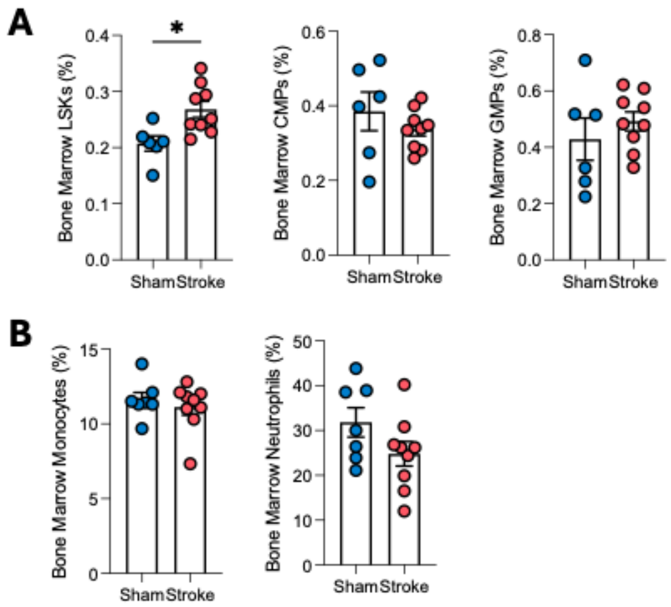

Bone marrow progenitor responses after stroke. (A) Quantification of LSKs, CMPs, and GMPs in femoral bone marrow at 24 h post-stroke. **(B)** Quantification of BM neutrophils and monocytes. Student's t-test. *$p<0.05$. n=6-9 per group.

Supplementary Figure 4

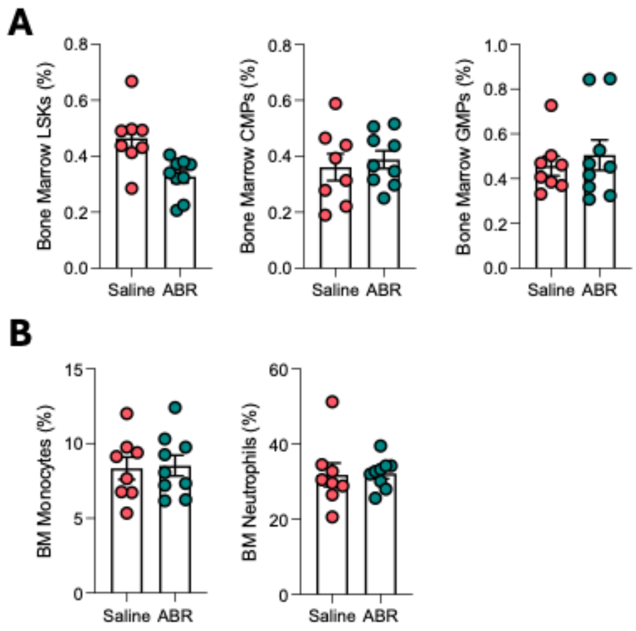

Bone marrow progenitor responses after ABR-215757. (A) Effect of ABR-215757 treatment on BM progenitor populations compared with vehicle. **(B)** Quantification of BM neutrophils and monocytes. n=8-9 per group.
